# Safety and efficacy of canine gonadal tissue-derived mesenchymal stem cells for early myxomatous mitral valve disease

**DOI:** 10.3389/fvets.2024.1404607

**Published:** 2024-10-02

**Authors:** Soyoung Jeung, Ju-Hyun An, Sung-Soo Kim, Hwa-Young Youn

**Affiliations:** ^1^VIP Animal Medical Center, Seoul, Republic of Korea; ^2^Laboratory of Veterinary Internal Medicine, Research Institute for Veterinary Science and College of Veterinary Medicine, Seoul National University, Seoul, Republic of Korea; ^3^Laboratory of Veterinary Emergency and Critical Care, Department of Veterinary Clinical Science, College of Veterinary Medicine, Kangwon National University, Chuncheon, Gangwon-do, Republic of Korea

**Keywords:** ACVIM stage B1, canine, early stage, gonadal tissue, heart disease, mesenchymal stem cell, myxomatous mitral valve disease, regenerative medicine

## Abstract

**Introduction:**

This study explored the potential efficacy and safety of therapy with mesenchymal stem cells (MSC) derived from gonadal tissue to address the early stage of myxomatous mitral valve disease (MMVD), the predominant cardiac condition in dogs.

**Methods:**

Sixteen dogs diagnosed with MMVD B1 were enrolled in this trial and assigned to either a control group (control group, *n* = 10) or a group that received MSC derived from gonadal tissue (treatment group, *n* = 6). In the treatment group, allogeneic MSC derived from gonadal tissue (1 × 10^6^ cells/kg) were intravenously administered at monthly intervals for five or more sessions. Data were compared at baseline and at the endpoint 1-year intervals. The efficacy was assessed using echocardiography, thoracic radiography, NT-proBNP, and the duration from B1 diagnosis to B2 transition to evaluate its effect on MMVD stage progression. Safety was evaluated through physical examinations, blood tests, imaging studies, and monitoring of adverse events.

**Results:**

After 1 year of observation, the control group exhibited deteriorating echocardiographic parameters, whereas the treatment group displayed no substantial differences between baseline and endpoint measurements. Notably, a statistically significant disparity was noted in the left atrial diameter (*p* < 0.05) and E-wave velocity (*p* < 0.05) between the two groups, indicating a favorable impact of MSC derived from the gonadal tissue on left atrial pressure. Additionally, in contrast to the control group, the treatment group demonstrated delayed progression to MMVD stage B2, enabling them to prolong their disease duration without requiring cardiac medication (*p* = 0.038). In quality of life (QoL) metrics following MSC treatment, appetite showed a statistically significant improvement, increasing from 4 to 4.83 (*p* < 0.05).

**Discussion:**

Treatment with gonadal tissue-derived MSCs significantly delayed MMVD stage progression, highlighting the broad potential of MSC derived from gonadal tissue for treating complex veterinary conditions.

## 1 Introduction

Chronic degenerative valve disease predominantly affects the mitral valve in small dogs and is the most common canine heart disease, accounting for approximately 75% of all cardiac cases (1–3). This degenerative disease causes leaflet thickening, fibrosis, and prolapse leading to blood regurgitation (4). Subsequent volume overload can result in morphological changes in the heart, eventually leading to heart failure and pulmonary edema (2).

Myxomatous mitral valve disease (MMVD) is categorized into four stages: Stage A diagnosing predisposed breeds without evident structural cardiac changes, Stage B for morphological cardiac remodeling, Stage C for clinical signs, and Stage D for resistance to standard treatments (3). MMVD stage B is characterized by the absence of clinical signs. Stage B is further divided into B1 and B2, with B1 indicating morphological changes that do not meet the clinical criteria and B2 indicating more severe morphological changes and mitral valve regurgitation, leading to hemodynamic issues (3). Stage B2 is defined by a murmur intensity grade of 3 or higher, a vertebral heart score exceeding 10.5, an LA:AO ratio of 1.6 or higher, and a left ventricular internal diameter in diastole of 1.7 or higher (3).

Approximately 30% of MMVD progress to heart failure (5). In the EPIC study, Boswood et al. recommended the administration of a single oral medication (pimobendan) at the MMVD B2 stage to prevent the onset of heart failure (6). Heart failure causes mild-to-severe clinical signs, including lethargy, anorexia, coughing, exercise intolerance, collapse, and breathing difficulty (3). Therapy for heart failure requires the coordinated use of multiple drugs, including diuretics, positive inotropic drugs, vasodilator drugs, angiotensin-converting enzyme inhibitors, beta-adrenergic blockers, or antiarrhythmic drugs (3, 7). Still, medications are the standard options to control clinical signs, but other supportive treatments can be attempted.

Mesenchymal stem cell therapy involves self-renewal, tissue regeneration, anti-inflammation, and immunomodulation (8–11). With the increase in the aging population among humans, there is a corresponding rise in the number of elderly animals in veterinary medicine. Consequently, there is an escalating demand for treatments addressing various intractable diseases, chronic conditions requiring long-term management, and complex health issues in elderly animals. Mesenchymal stem cells (MSCs) are emerging as a promising therapeutic solution for these challenges. Mesenchymal stem cells can be derived from various tissues, such as the bone marrow, adipose tissue, and placenta (12). However, the additional surgical procedures necessary to obtain the donor source can lead to significant issues regarding cost, time, and health. Additionally, cryopreserving an animal’s own mesenchymal stem cells at a young age offers the advantage of potentially receiving MSC therapy in the future with reduced immunogenic side effects. Therefore, we investigated the potential of repurposing gonadal tissue, which is typically discarded during neutering procedures, as an innovative source of MSC therapy.

In veterinary medicine, it is primarily used to treat conditions in dogs, such as musculoskeletal, neuromuscular, and renal diseases. In cats, significant therapeutic effects have been reported in cases of ischemic acute kidney injury, chronic kidney disease, gingivostomatitis, experimental asthma, and chronic enteropathy (13, 14). Various veterinary medicine studies have explored mesenchymal stem cell therapies for various heart conditions. Intracoronary allogeneic cardiosphere-derived stem cells initially show partial improvement in dogs with dilated cardiomyopathy (DCM). Fractional shortening (FS%), which is a marker of cardiac function, continued to decrease in the control group. In contrast, mesenchymal stem cell therapy robustly preserved FS% in the treatment group, comprising responders and non-responders to cardiosphere-derived stem cell treatment. Similar trends were noted for percent wall thickness, which also serves as a marker of cardiac function (15). In cases of chronic Chagas cardiomyopathy in canines, significant improvement in cardiac function, as indicated by the peak velocity of aortic flow, was observed after the implantation of autologous bone marrow-derived mesenchymal stem cells into the right and left coronary arteries (16). Studies on MSC therapy for canine chronic valvular heart disease have mostly focused on patients with MMVD stages C and D. In addition, Petchdee et al. intravenously transplanted dogs with allogeneic puppy deciduous teeth stem cells (pDSCs). Compared with the control group receiving standard treatment, the MSC therapy group exhibited significant improvements in left ventricular ejection fraction, left atrium and aortic root ratio, ACVIM stage, and quality of life scores up to 60 days after MSC administration (17). In contrast, Yang et al. found that intravenous injection of Wharton jelly derived mesenchymal stem cells into dogs with congestive heart failure secondary to MMVD did not result in significant improvements in cardiac function, prolonged survival time, or therapeutic effects of diuretic dosing (18). This study aimed to assess the effectiveness of mesenchymal stem cell therapy in canines diagnosed with early stage heart disease (MMVD ACVIM stage B1).

## 2 Materials and methods

### 2.1 Study population

Client-owned patients with early stage MMVD (Stage B1) who visited the VIP Animal Medical Center were evaluated at yearly intervals. The patients were divided into gonadal tissue-derived MSC treatment (*n* = −6) and control (*n* = 10) groups. Patients’ medical records were reviewed using an electronic charting program (E-friends, pnV Co., Ltd., Seoul, Korea). This retrospective study was approved by the Institutional Animal Care and Use Committee (IACUC) of the VIP Animal Medical Center (protocol no. VIP-0006-SC).

#### 2.1.1 Inclusion and exclusion criteria

From March 2019 to December 2022, client-owned patients with early-stage MMVD (Stage B1) who were diagnosed at the VIP Animal Medical Center were divided into two groups. The “control group” (*n* = 10) consisted of patients who were only monitored, while the “MSC group” (*n* = 6) included patients who started gonadal tissue-derived MSCs therapy within 1 year of diagnosis. However, patients with other serious diseases or those who did not visit the hospital within the evaluation period were excluded from the final analysis.

### 2.2 Tissue collection and cell preparations

We obtained female gonadal tissues (ovaries) that were discarded during neutering surgery at the VIP Animal Medical Center with the consent of the owners. Donors were thoroughly screened for infectious diseases and showed no abnormal findings on blood or imaging examinations. This study was approved by the Institutional Animal Care and Use Committee (IACUC) of the VIP Animal Medical Center (Protocol no. VIP-0004-SC). Mesenchymal stem cells were isolated as follows. The tissue derived from the donor was extracted from a sterile container using sterilized forceps and transferred to a tube containing 20 mL of Dulbecco’s phosphate-buffered saline (DPBS; Thermo Fisher Scientific, Massachusetts, USA) for thorough washing. This process was repeated three times. After washing, the tissue was transferred to a fresh Petri dish and meticulously cut into approximately 1–2 mm sections using sterilized scissors. Subsequently, it underwent enzymatic digestion in a 1% collagenase diluted solution (Sigma-Aldrich, St. Louis, USA) along with MEM-alpha medium (*α*-MEM; Sigma-Aldrich, St. Louis, USA) for 2 h at 37°C in a water bath. Subsequently, the cell suspension was first filtered using a 100 μm cell strainer (from SPL Life Sciences Co., Gyeonggi-do, Korea) followed by a 40 μm strainer. The resulting cell suspension was centrifuged at 700 *g* for 10 min at room temperature. The pellet was washed at least three times with DPBS. Subsequently, the cells were resuspended in 1 mL of culture medium. The culture medium consisted of minimum essential medium *α* (MEM-α) supplemented with 10% fetal bovine serum (FBS) and 1% penicillin/streptomycin. The resuspended cells were then transferred to a 100 mm culture dish containing 9 mL of culture medium and incubated at 37°C with 5% CO_2_. The culture medium was changed daily until non-MSC impurities were eliminated, after which it was changed every 2 days.

### 2.3 Cell characterization

To characterize the MSCs, immunophenotyping were performed via flow cytometry (BD Accuri^™^ C6 Plus, BD Biosciences, Franklin Lakes, New Jersey, USA). Most cells were positive for CD 29 (FITC) and CD 44 (FITC); however, a few were negative for CD90 (PE). FITC-conjugated CD29 (antibody clone MEM-101A; Invitrogen, Massachusetts, USA) and FITC-conjugated CD44 (antibody clone IM7; Invitrogen) were used as canine MSCs. PE-conjugated CD90 (antibody clone YKIX337.217; Invitrogen, Carlsbad, CA, USA) was used for canine MSCs.

MSCs were differentiated using commercial kits for 2 weeks and identified via staining for adipocytes (with Oil Red O), osteocytes (with Alizarin Red S), and chondrocytes (with Alcian blue) (StemPro Adipogenesis, Osteogenesis, and Chondrogenesis Differentiation kits, Thermo Fisher Scientific, Massachusetts, USA).

### 2.4 Mesenchymal stem cell therapy

Allogeneic gonadal tissue-derived MSCs (1 × 10^6^ cells/kg) were intravenously administered at monthly intervals for five or more sessions. Intravenous (IV) injection was administered for approximately 20–30 min at a rate of 0.5–1 mL/min or less, and the syringe was gently rolled to ensure that the diluted cells were fully dispersed before the injection. The dosage and treatment regimen for stem cell therapy were determined based on prior studies (17, 19–21).

To mitigate concerns about potential adverse immune reactions, we administered antihistamine injections prior to treatment, following recommendations from previous literature (22, 23). Before allogeneic mesenchymal stem cell therapy, Chlorpheniramine maleate 0.2 mg/kg, SC (Histamine, SAMU MEDIAN Co., Ltd., Seoul, Korea) were administered 30 min before treatment to prevent immune reactions. Fluid therapy was administered for 30 min before and after cell therapy. The main types of fluid used was 0.9% normal saline and the speed was maintained at 2.5 mL/kg/h.

Patients were monitored for over an hour for hypersensitivity reactions before returning home. The patient returned for the evaluation of treatment response or side effects according to the monitoring schedule.

### 2.5 Assessment of MSC therapy response

Patients in the mesenchymal stem cell therapy group were evaluated by comparing data from the baseline to the endpoint (1 year after treatment). The control group comprised patients diagnosed with MMVD within 1 year who were evaluated by comparing the data from regular heart monitoring examinations at yearly intervals. On the day of MSC therapy, the patients’ body temperatures were monitored. Weight changes were assessed at yearly intervals. To evaluate the safety of MSC treatment, we monitored patient status by comparing pre- and post-treatment results, including complete blood counts, serum chemistry, and electrolytes. Echocardiography results were analyzed in the MSC treatment group before the initiation of mesenchymal stem cell therapy (baseline) and at the 1-year follow-up assessment (endpoint). The control group was compared at 1-year mark after diagnosis in patients with stage B1 MMVD. Patients with MMVD were diagnosed with stage B upon the identification of cardiac remodeling without clinical signs. Specifically, stage B2 was diagnosed if an left atrium: aorta ratio of 1.6 or higher and a left ventricular internal diameter in diastole of 1.7 or higher were confirmed by more than three imaging specialists. If these criteria were not met, the patient was diagnosed with stage B1. Additionally, the prescription and timing of pimobendan, a medication indicated for MMVD stage B2, were reviewed through the patient’s medical records.

We conducted quality of life (QoL) evaluations in both the experimental and control groups using the Canine Health-Related Quality of Life Survey (CHQLS-21) as a reference (24). Each item scored from 1 to 5, where higher scores indicate more positive outcomes.

### 2.6 Statistical analysis

Statistical analyses were performed using GraphPad Prism Version 9 (GraphPad Inc., La Jolla, CA, USA). Numerical data were presented as the mean ± standard deviation. On echocardiography, baseline and endpoint values were compared at yearly intervals for each group using paired t-, nonparametric, and Wilcoxon tests. The comparison between the MSC treatment and control groups involved paired t-, nonparametric, and Mann–Whitney test. *p*-values are indicated by *p < 0.05 and **p < 0.01 and were considered statistically significant.

## 3 Results

### 3.1 MSC characterization and differentiation

**Figure 1:**
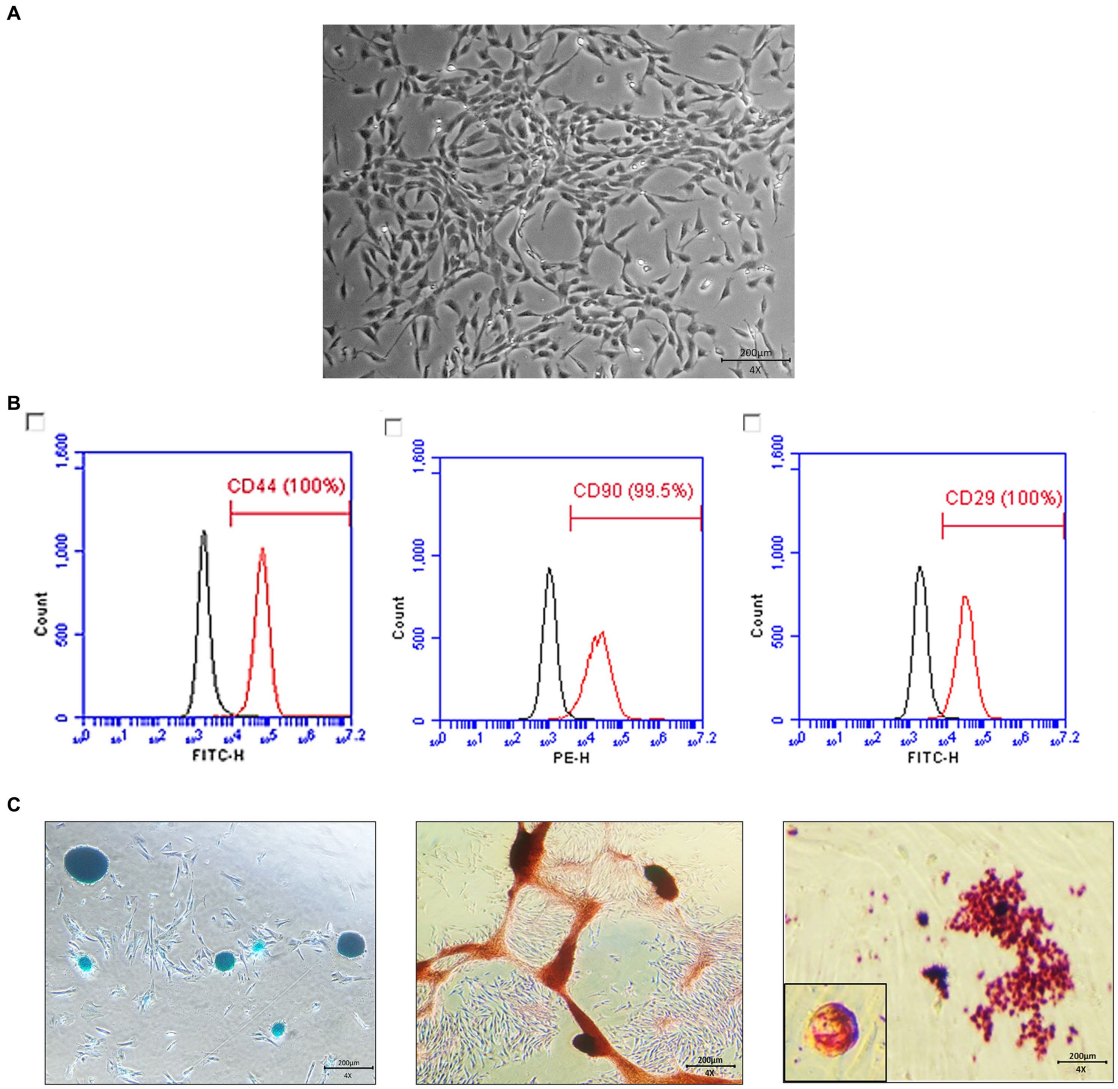
Characterization and differentiation of canine gonadal tissue-derived mesenchymal stem cells (MSCs). The morphology of canine gonadal tissue-derived MSCs under light microscopy, where confluent cells exhibit the typical spindle-like shape characteristic of MSCs with a scale bar representing 200 μm **(A)**. The expression of surface markers of canine gonadal tissue-derived MSCs is depicted, with the left, middle, and right panels corresponding to the expression of CD44, CD90, and CD29, respectively, as measured using flow cytometry **(B)**. The chondrogenic, osteogenic, and adipogenic differentiation of canine gonadal tissue-derived MSCs, with the left panel showing chondrocytes differentiated from MSCs stained with alcian blue, the middle panel displaying osteoblasts originated from MSCs stained with alizarin red S, and the right panel exhibiting adipocytes differentiated from MSCs stained with Oil red-O **(C)**.

The gonadal tissue-derived MSCs exhibited a spindle-shaped, fibroblast-like morphology (Figure 1A). Canine gonadal MSCs were stained for essential MSC-positive markers (CD29, CD44, and CD90) using various antigens. The expression levels of these markers consistently exceeded 95% (Figure 1B). The MSCs were induced to differentiate into chondrocytes, osteoblasts, and adipocytes, and the staining results were positive (Figure 1C).

### 3.2 Study animals

Data were collected from 16 dogs with MMVD (ACVIM stage B1), with six dogs in the MSC therapy group and 10 in the control group (Table 1). The median ages of the MSC therapy and control groups was 12 and 13 years, respectively, with no statistically significant difference (*p* = 0.9257). Similarly, the weights of the two groups were not significantly different (*p* = 0.651) (Table 2).

**Table 1:** Patients’ information.

Patient	Group	Breed	Age	Sex	Body weight (kg)	MMVD stage	Progression to MMVD stage B2	Concurrent disease
A1	MSC	Long coat chihuahua	11	FS	4.26	B1	Progression	CKD1
A2	MSC	Welsh Corgis	14	FS	11.8	B1	Not yet	Cushing disease, DJD, CDS, IVDD, DJD, TC
A3	MSC	Pomeranian	13	MN	4	B1	Not yet	MPL, IVDD, DJD, TC
A4	MSC	Mixed	11	FS	6.2	B1	Progression	Cataracts, UB calculi
A5	MSC	Maltese	20	FS	2.5	B1	Progression	CKD, DJD, TC, MPL
A6	MSC	Long coat chihuahua	10	MN	2.71	B1	Not yet	TC, Elongated soft palate, UB calculi
B1	Control	Mixed	11	MN	5.5	B1	Progression	GBS
B2	Control	Maltese	15	FS	3.1	B1	Not yet	CKD1, DJD, Cholestasis, Hydrocephalus, Geriatric vestibular disease
B3	Control	Poodles	14	FS	4.12	B1	Progression	CCLR
B4	Control	Mixed	13	MN	5.2	B1	Progression	TC, Hyperlipidemia, Hypothyroidism
B5	Control	Maltese	12	MN	5.2	B1	Not yet	Hyperlipidemia, DJD, Cataracts, MPL
B6	Control	Maltese	12	MN	4	B1	Progression	Urolithiasis, Atopic dermatitis
B7	Control	Shih Tzu	16	FS	4.94	B1	Progression	Hypothyroidism, TC
B8	Control	Pomeranian	9	MN	3.8	B1	Not yet	CKD1
B9	Control	Poodles	10	FS	7	B1	Not yet	MPL
B10	Control	Mixed	14	MN	6	B1	Not yet	Renal calculi, UB calculi, GBM

**Table 2:** Age and body weight at baseline.

Index	MSC group	Control group	*p*-value
Median age at baseline (Median, range)	12 (10–20)	13 (9–16)	–
Mean age at baseline (Mean ± SD, years)	13.17 ± 3.66	12.60 ± 2.11	0.9257
Mean body weight at baseline (Mean ± SD, kg)	5.25 ± 3.17	4.89 ± 1.10	0.651

The MSC therapy group consisted of four spayed females and two neutered males, whereas the control group included four spayed females and six neutered males. In the MSC therapy group, the average time from diagnosis of MMVD ACVIM stage B1 to the initiation of mesenchymal stem cell therapy was 395.17 ± 257.00 days.

### 3.3 Echocardiographic analysis

**Figure 2:**
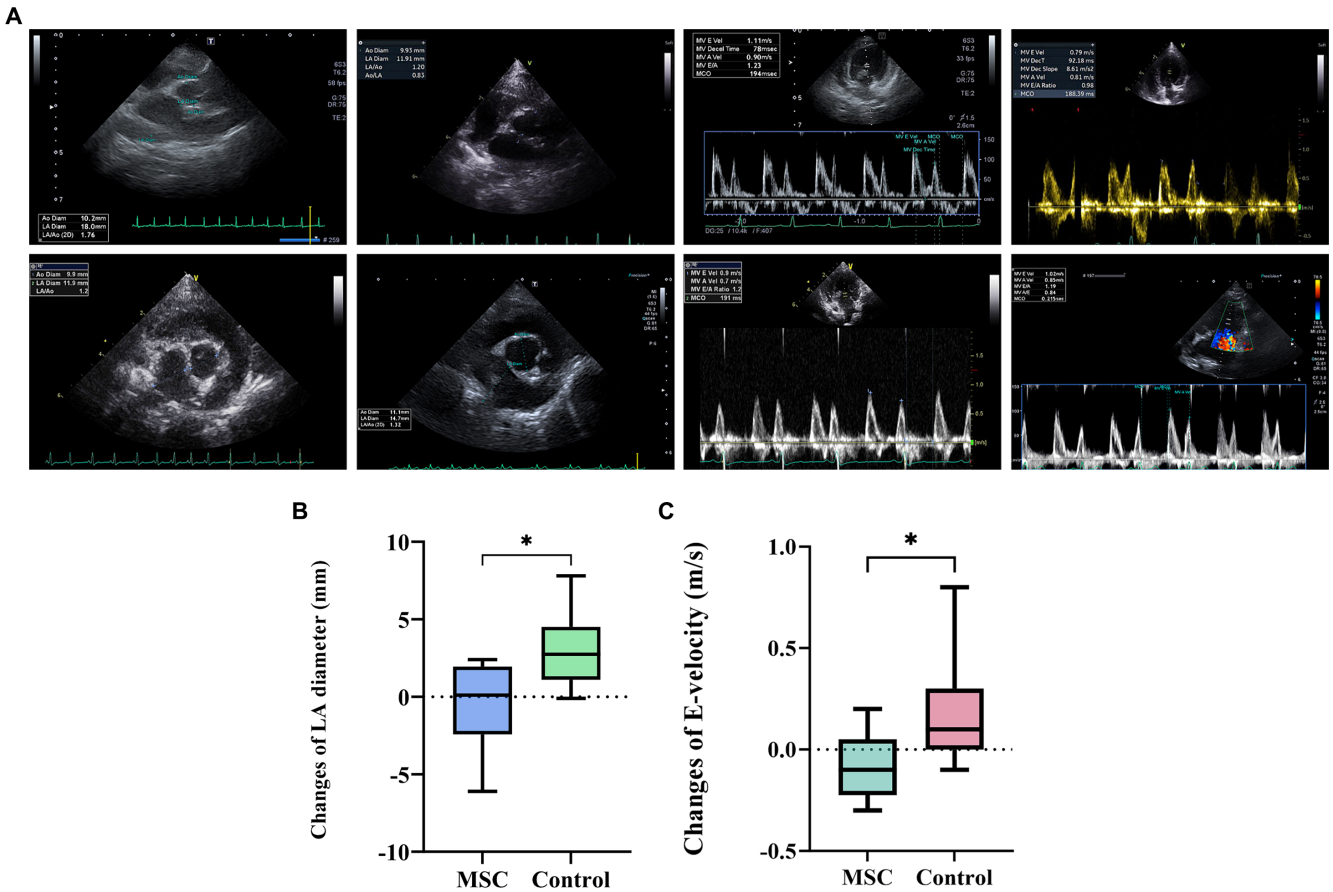
Comparative analysis of echocardiographic changes in the MSC and control groups. The comparison of changes in echocardiography between the endpoint and baseline in the MSC and control groups **(A)**. Patient A1 demonstrated improvement from 18 mm to 11.9 mm in LA diameter before (baseline) and after (endpoint) stem cell therapy, respectively, with the E-peak decreasing from 1.1 m/s to 0.8 m/s post-treatment. In contrast, patient B3, part of the control group, exhibited an increase in LA diameter from 11.9 mm to 14.7 mm after 1 year, with the E-velocity rising from 0.9 m/s to 1 m/s. The changes in left atrium diameter between endpoint and baseline after canine gonadal tissue-derived MSCs therapy, showing differences of 0.48 ± 2.86 mm in the MSC group and 2.97 ± 2.38 mm in the control group, indicating a statistically significant difference (*p* < 0.05) **(B)**. The changes in E-velocity between endpoint and baseline after canine gonadal tissue-derived MSCs therapy, with the E-velocity changes of −0.08 ± 0.16 m/s in the MSC group and 0.26 ± 0.30 m/s in the control group, showing a statistically significant difference between them (*p* < 0.05) **(C)**. *p < 0.05.

At baseline, there was no statistically significant difference observed in echocardiographic and radiographic parameters between the MSC group and the control group (Table 3). Echocardiographic parameter changes were calculated by subtracting baseline measurements from endpoint measurements. The control group demonstrated a statistically significant deterioration in LA (*p* = 0.0078), LA/AO ratio (*p* = 0.0117), LVIDDN (*p* = 0.0098), E-velocity (*p*-value = 0.0391), and VHS (*p* = 0.0312) from the baseline to the endpoint at yearly intervals. In contrast, the MSC therapy group exhibited no significant changes in the echocardiographic measurements during the same period. Although not statistically significant, the MSC group showed improved LA and E-velocity (Table 4). In Figure 2A, echocardiographic features of the MSCs and control groups are presented. One patient in the MSC group exhibited a decrease in the LA from 18 mm to 11.9 mm after mesenchymal stem cell treatment, with an improved LA/AO ratio from 1.8 to 1. Additionally, the E-peak improved from 1.1 m/s to 0.8 m/s. For a patient in the control group, the LA increased from 11.9 mm to 14.7 mm over a 1-year interval, with the LA/AO ratio progressing from 1.2 to 1.3. Moreover, the E-peak worsened from 0.9 m/s to 1 m/s. Comparisons were established by subtracting the baseline values from the endpoint values for each parameter, with echocardiographic evaluations conducted at yearly intervals (Table 5). Changes in values were calculated by subtracting the baseline measurements from the endpoint measurements. For LA diameter, differences were observed with −0.48 ± 2.86 mm in the MSC group and 2.97 ± 2.38 mm in the control group (*p* < 0.05) (Figure 2B). In terms of E-velocity, changes were − 0.08 ± 0.16 m/s in the MSC group and 0.26 ± 0.30 m/s in the control group (*p* < 0.05) (Figure 2C).

**Table 3:** Comparisons echocardiographic and radiographic parameters between the MSCs and control groups at baseline.

Index	MSC			Control			*p-*value
	Mean	SD	*N*	Mean	SD	*N*	
LA (mm)	17.10	4.36	6	15.24	3.16	9	0.4559
LA/Ao	1.37	0.27	6	1.26	0.20	10	0.6773
LVIDd (mm)	21.27	6.03	6	22.91	3.99	9	0.7756
LVIDd inc%	−12.60	20.49	6	−5.01	13.33	10	0.4278
LVIDd/Ao	1.72	0.63	6	1.92	0.25	10	0.1111
LVIDdN	1.32	0.35	6	1.28	0.32	10	0.8059
E vel (m/s)	0.73	0.23	6	0.81	0.21	10	0.6518
E/E′	12.07	6.42	6	13.59	4.78	10	0.4278
E/IVRT	1.08	0.47	5	1.33	0.58	9	0.5160
EDVI (vet teich.)	29.83	16.53	6	37.85	15.46	10	0.3001
E/A	1.12	0.36	6	1.15	0.18	10	0.9813
E′/A′	0.97	0.46	6	0.74	0.14	10	0.4100
IVRT	65.00	14.05	5	67.00	15.18	9	0.9730
SF (%)	44.80	11.27	6	48.66	11.21	10	0.5097
ESVI (vet teich.)	4.87	3.31	6	7.26	7.28	10	0.5808
LVIMP (Tei index)	0.75	0.51	5	0.58	0.44	9	0.7514
LV S′max	8.08	2.36	6	9.43	3.04	9	0.7489
AV flow (m/s)	1.05	0.23	6	1.16	0.24	10	0.4046
PV flow (m/s)	1.00	0.41	6	0.92	0.23	10	0.9819
MR vel (m/s)	5.20	1.00	5	5.04	1.27	8	0.8765
AR vel (m/s)	0	0	5	0.52	1.16	5	>0.9999
PR vel (m/s)	1.63	1.46	3	0.53	0.92	3	0.400
TR vel (m/s)	1.83	1.60	3	1.80	1.57	3	>0.9999
VHS	10.08	0.73	6	9.78	0.28	9	0.3700
VLAS	2.12	0.29	6	2.09	0.19	9	0.9744

**Table 4:** Changes in echocardiographic and radiographic parameters between endpoint and baseline in respective groups.

Index	MSC (Mean ± SD)			Control (Mean ± SD)		
	Baseline	Endpoint	*p*-value	Baseline	Endpoint	*p*-value
MR (m/s)	4.33 ± 2.10	4.60 ± 2.06	0.8750	4.48 ± 1.94	4.97 ± 1.99	0.2109
LA (mm)	17.10 ± 3.98	16.62 ± 4.43	0.8438	15.24 ± 2.98	17.96 ± 2.72	0.0078
LA/Ao	1.37 ± 0.24	1.37 ± 0.23	0.4375	1.26 ± 0.19	1.52 ± 0.26	0.0117
LVIDd (mm)	21.27 ± 5.51	21.52 ± 5.86	0.7500	22.91 ± 3.76	24.26 ± 4.14	0.0664
LVIDdN	1.32 ± 0.32	1.35 ± 0.28	0.7500	1.28 ± 0.30	1.57 ± 0.21	0.0098
E-velocity (m/s)	0.73 ± 0.21	0.65 ± 0.17	0.3750	0.81 ± 0.20	1.01 ± 0.31	0.0391
SF (%)	44.80 ± 10.29	43.78 ± 7.82	>0.9999	48.66 ± 10.63	50.61 ± 11.67	0.8457
VHS	10.08 ± 0.67	10.28 ± 0.56	0.2500	9.78 ± 0.27	10.21 ± 0.43	0.0312
VLAS	2.12 ± 0.27	2.20 ± 0.19	0.5000	2.09 ± 0.18	2.17 ± 0.18	0.5000

**Table 5:** Comparisons echocardiographic and radiographic parameters change between endpoint and baseline in the MSC and control groups.

Index	MSC (Endpoint-baseline)			Control (Endpoint-baseline)			*p-*value
	Mean	SD	*N*	Mean	SD	*N*	
LA (mm)	−0.48	2.86	6	2.97	2.38	10	0.0335
LA/Ao	0.00	0.36	6	0.29	0.30	10	0.4339
LVIDd (mm)	0.25	5.30	6	1.63	2.11	9	0.9766
LVIDd inc%	1.15	22.77	6	8.96	9.53	10	>0.9999
LVIDd/Ao	0.10	0.55	6	0.23	0.18	10	0.9349
LVIDdN	0.03	0.31	6	0.37	0.22	10	0.1856
E vel (m/s)	−0.08	0.16	6	0.26	0.30	10	0.0357
E/E′	2.97	8.61	6	4.24	3.75	10	0.8749
E/IVRT	0.00	0.54	6	0.32	0.78	9	0.5099
EDVI (vet teich.)	−6.85	19.96	6	2.79	16.99	10	0.6164
E/A	−0.18	0.41	6	0.00	0.18	10	0.3139
E′/A′	−0.25	0.33	6	0.13	0.26	10	0.1206
IVRT	25.83	31.37	6	−0.83	16.57	9	0.1205
SF (%)	−1.02	4.82	6	1.24	18.46	10	0.7925
ESVI (vet teich.)	1.32	1.96	6	−0.73	10.45	10	0.9794
LVIMP (Tei index)	0.09	0.68	6	−0.09	0.63	9	0.6070
LV S′max	−0.25	2.83	6	−1.72	2.77	9	0.8639
AV flow (m/s)	−0.05	0.18	6	−0.06	0.18	10	0.7055
PV flow (m/s)	−0.02	0.38	5	−0.01	0.14	10	0.9863
MR (m/s)	0.27	0.80	6	0.38	0.76	9	0.5900
AR vel (m/s)	0.48	1.08	6	0.07	1.37	9	0.6176
PR vel (m/s)	−0.64	1.09	5	0.25	0.59	8	0.1305
TR vel (m/s)	−0.13	0.26	6	0.26	1.38	9	0.2841
VHS	0.20	0.30	6	0.47	0.37	10	0.4378
VLAS	0.08	0.18	6	0.06	0.18	10	0.3258

### 3.4 Comparisons of changes in NT-proBNP between the endpoint and baseline in MSC treatment and control groups

**Figure 3:**
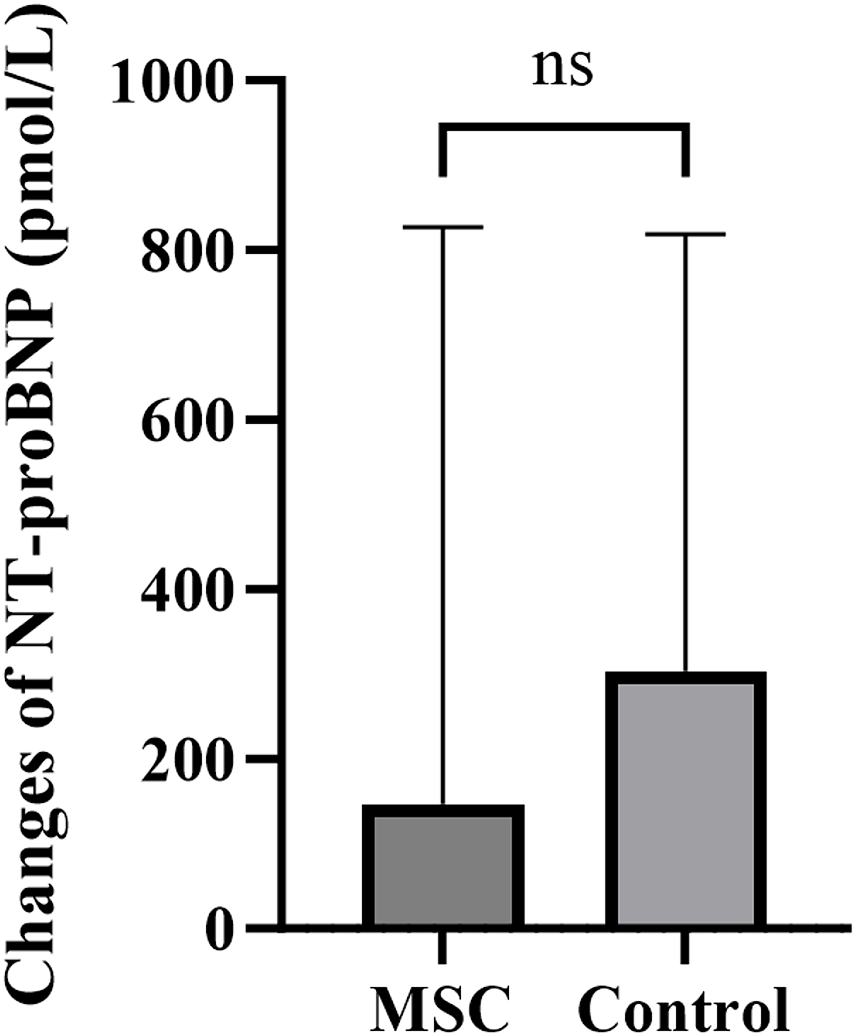
The changes in NT-proBNP between the endpoint and baseline after canine gonadal tissue-derived MSC therapy. The mean changes in serum NT-proBNP were 146.80 ± 608.72 pmol/L and 304.14 ± 476.75 pmol/L in the MSC and control groups, respectively, with no statistically significant difference between them.

NT-proBNP measurements were performed annually at baseline and endpoint. The change in values was calculated by subtracting the baseline measurements from the endpoint measurements. The mean changes in serum NT-proBNP were 146.80 ± 608.72 pmol/L and 304.14 ± 476.75 pmol/L for the MSC and control groups, respectively. No significant differences were observed between the control and MSC groups (Figure 3).

### 3.5 Analyzing the progression of MMVD ACVIM stage using the Kaplan–Meier curve

**Figure 4:**
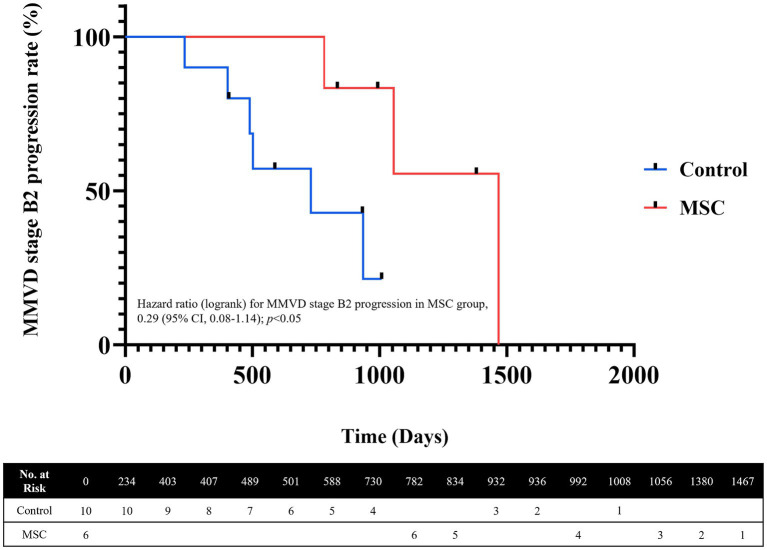
Analysis of MMVD ACVIM stage progression using Kaplan–Meier Curves. The median progression duration was 730 days for the control group and 1,467 days for the MSC group. The significance level for MMVD stage progression analysis (*p =* 0.038).

Among the six dogs in the MSC therapy group, three progressed from MMVD B1 to B2 during the 1-year monitoring period. In contrast, among the 10 dogs in the control group that were monitored for 1 year, five progressed from MMVD B1 to B2 (Table 6). The mean duration from the B1 diagnosis to the transition to B2 was 1101.67 ± 344.78 days in the MSC therapy group and 548.83 ± 248.59 days in the control group. The median progression duration of MMVD (from B1 to B2) was 730 days in the control group and 1,467 days for the MSC group, respectively (*p =* 0.038) (Figure 4).

**Table 6:** Medication use in the MSC and control groups during the evaluation period.

Patient	Group	Breed	Age	MMVD stage	Progression to MMVD stage B2	Medications
A1	MSC	Long coat chihuahua	11	B1	Progression	Pimobendan
A2	MSC	Welsh Corgis	14	B1	Not yet	–
A3	MSC	Pomeranian	13	B1	Not yet	–
A4	MSC	Mixed	11	B1	Progression	Pimobendan
A5	MSC	Maltese	20	B1	Progression	Pimobendan
A6	MSC	Long coat chihuahua	10	B1	Not yet	–
B1	Control	Mixed	11	B1	Progression	Pimobendan
B2	Control	Maltese	15	B1	Not yet	–
B3	Control	Poodles	14	B1	Progression	Pimobendan
B4	Control	Mixed	13	B1	Progression	Pimobendan
B5	Control	Maltese	12	B1	Not yet	–
B6	Control	Maltese	12	B1	Progression	Pimobendan
B7	Control	Shih Tzu	16	B1	Progression	Pimobendan
B8	Control	Pomeranian	9	B1	Not yet	–
B9	Control	Poodles	10	B1	Not yet	–
B10	Control	Mixed	14	B1	Not yet	–

### 3.6 Quality of life scoring evaluation

**Figure 5:**
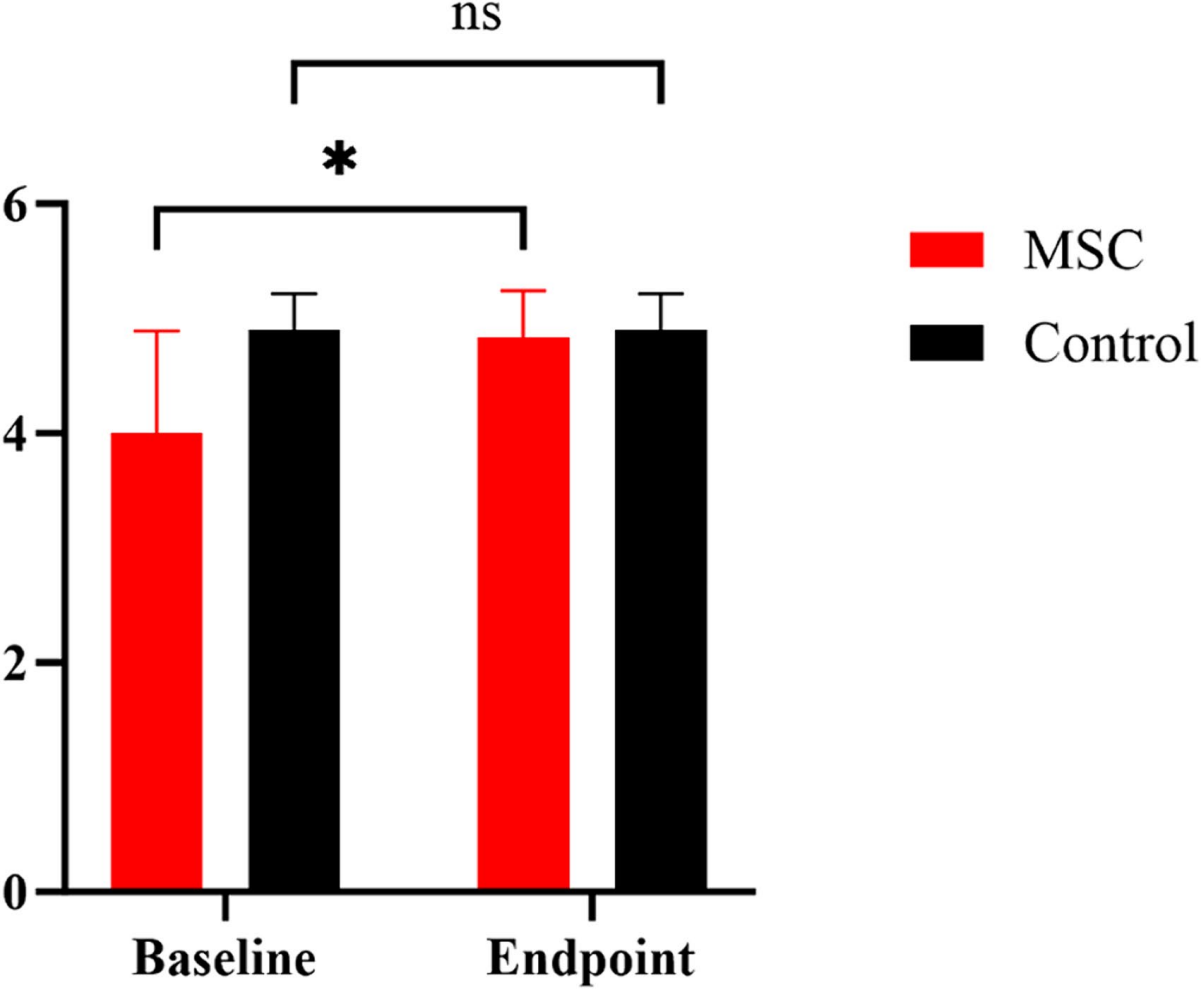
Comparison of MMVD stage B1 patients’ appetite between MSC therapy and control groups at baseline and endpoint. Quality of life (QoL) metrics were evaluated both before and after mesenchymal stem cell (MSC) treatment. Our results demonstrated a significant change in appetite rising from 4 to 4.83 in the MSC group pre- and post-treatment (*p* < 0.05). *p < 0.05.

**Figure 6:**
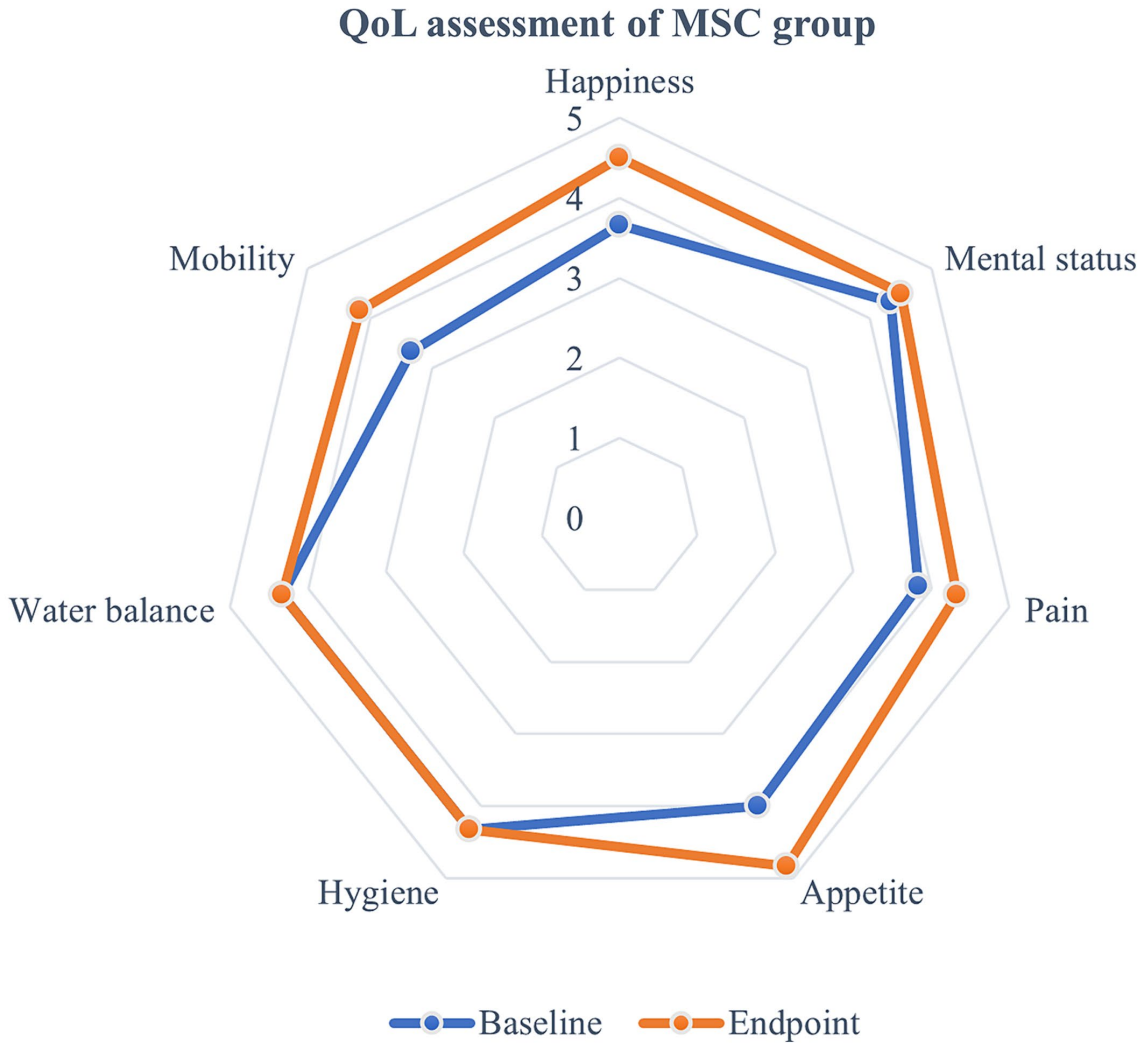
Evaluation of quality of life following MSC therapy in MMVD stage B1 patients. Quality of life (QoL) metrics were assessed before and after MSC treatment, revealing improvements following the therapy. Happiness increased from an average of 3.67 at baseline to 4.5 at the endpoint, while mental status improved from 4.33 to 4.5. Pain scores also rose from 3.83 to 4.33, and appetite significantly increased from 4 to 4.83 (*p* < 0.05). Although other QoL parameters such as hygiene, water balance, and mobility showed some changes, with mobility improving from 3.33 to 4.17, these changes were not statistically significant. The overall QoL score rose from 27.83 at baseline to 31 at the endpoint, indicating an enhancement in the patients’ quality of life after MSC therapy.

Quality of life (QoL) metrics were assessed both before and after mesenchymal stem cell (MSC) treatment, demonstrating statistically significant improvements post-treatment. Happiness increased from an average of 3.67 at baseline to 4.5 at the endpoint, and mental status improved from 4.33 to 4.5. Pain levels decreased, with scores rising from 3.83 to 4.33, and appetite showed a statistically significant improvement, increasing from 4 to 4.83 (*p* < 0.05) (Figure 5). Although other QoL parameters, such as hygiene, water balance, and mobility, also exhibited changes—mobility, for instance, improved from 3.33 to 4.17—these changes were not statistically significant (Figure 6). The overall QoL score increased from 27.83 at baseline to 31 at the endpoint, suggesting an improvement in the patients’ quality of life following MSC therapy. In the control group, no statistically significant differences were observed in the QoL assessment items over the course of 1 year.

### 3.7 Prescription of medication for MMVD

During the assessment period, pimobendan was prescribed to patients progressing to MMVD stage B2 in both groups (Table 6).

### 3.8 Safety analysis of MSCs therapy

**Figure 7:**
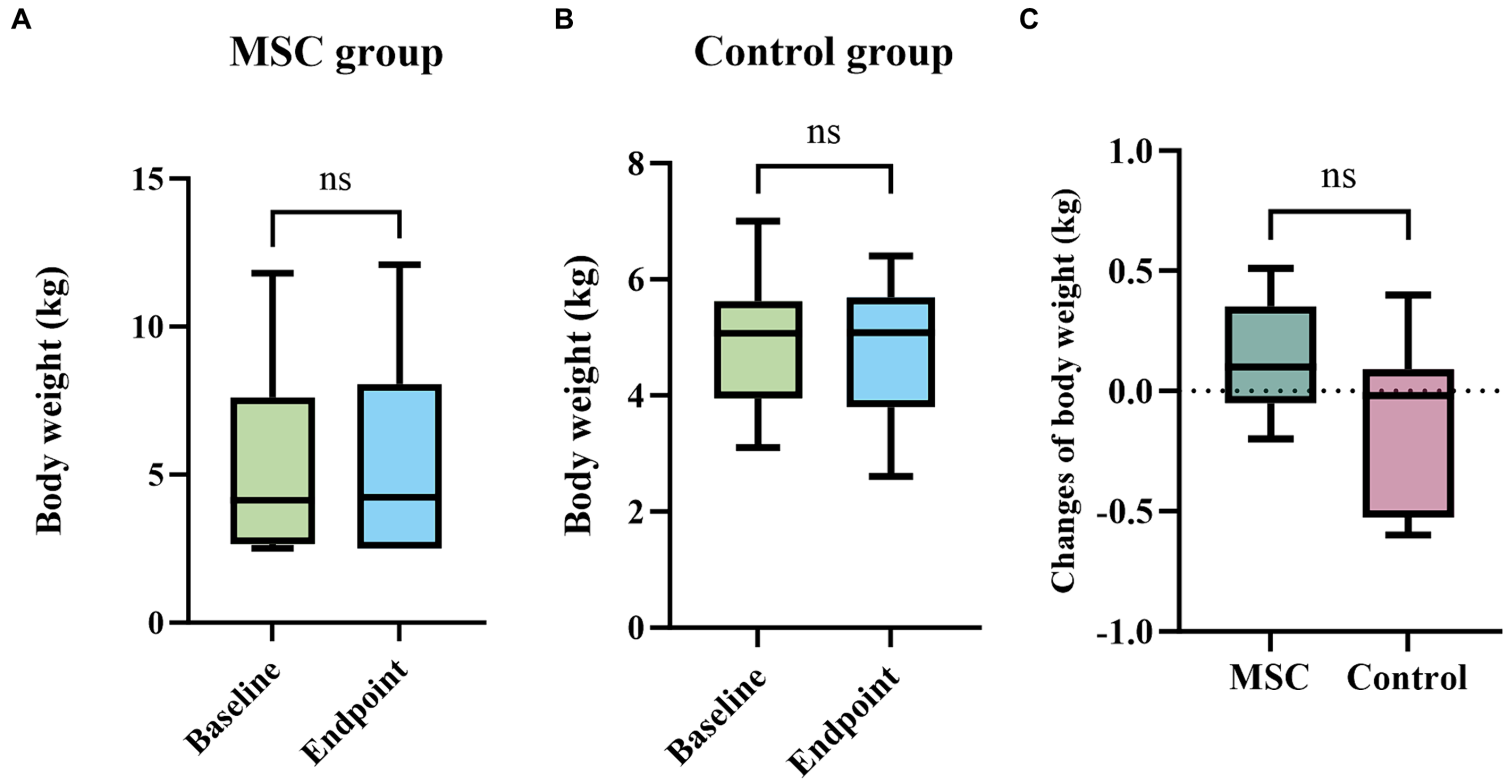
Assessment of stem cell safety with weight changes over 1 year. In both the MSC therapy and control groups, weight changes over the 1-year period were minimal, with the MSC group showing a slight increase **(A)** and the control group showing a slight decrease **(B)**. These changes were not statistically significant within each group **(A,B)** or between the groups **(C)**.

Weights were measured at yearly intervals in the MSC therapy group. At baseline, the weight was recorded as 5.25 ± 3.17 kg, and at the endpoint, it was 5.38 ± 3.32 kg, indicating a weight increase over the 1-year period. In the control group, the weights at baseline and endpoint were 4.89 ± 1.10 kg and 4.76 ± 1.17 kg, respectively, showing a weight loss trend over the year. However, there was no statistically significant difference in weight changes between baseline and endpoint within each group (MSC therapy group, *p* = 0.3125; control group, *p* = 0.3672) (Figures 7A,B). There was also no statistically significant difference in weight changes (endpoint-baseline) between the MSC therapy (0.14 ± 0.22 kg) and control groups (0.00 ± 0.25) (*p* = 0.1369) (Figure 7C). In the MSC therapy group, blood test results for complete blood count and serum chemistry (glucose, blood urea nitrogen, creatinine, ALP, ALT, total protein, albumin, globulin, albumin/globulin ratio, total bilirubin, GGT, total cholesterol, phosphorus, Amylase, Lipase, AST, and TG), and electrolytes (Na^+^,K^+^,Ca^2+^, and Cl^−^) were compared at baseline and short-term (3 months) and long-term (12 months) intervals. No statistically significant differences were observed between the short- and long-term blood test results before and after mesenchymal stem cell treatment (Table 7). During the monitoring period in the MSC therapy group, thoracic radiography and abdominal ultrasonography revealed no evidence of tumor formation. No short- or long-term adverse reactions were observed after the mesenchymal stem cell therapy.

**Table 7:** Short- and long-term blood analysis changes following stem cell treatment.

Mean ± SD						*p*-value	
Blood analysis	Category	Baseline	Short-term	Long-term	Unit	Baseline-short-term	Baseline-long-term
CBC	Hematocrit	48.23 ± 4.17	45.95 ± 3.27	46.70 ± 3.85	%	0.4352	0.675
	WBC	8.01 ± 2.46	8.25 ± 3.04	9.02 ± 2.64	K/μL	0.9724	0.6237
	Neutrophils	5.50 ± 1.86	5.68 ± 2.03	6.23 ± 2.12	K/μL	0.9774	0.7109
	Lymphocytes	1.63 ± 0.57	1.70 ± 0.83	1.71 ± 0.75	K/μL	0.8288	0.8151
	Monocytes	0.45 ± 0.13	0.47 ± 0.17	0.54 ± 0.11	K/μL	0.982	0.4893
	Platelets	344.17 ± 128.60	334.17 ± 216.13	374.83 ± 153.84	K/μL	0.953	0.6474
Serum-chemistry	Glucose	104 ± 10.49	97.17 ± 6.85	96.5 ± 13.4	mg/dL	0.4109	0.349
	BUN	15.92 ± 10.47	20.13 ± 14.49	21 ± 20.84	mg/dL	0.5041	0.3805
	Creatinine	0.98 ± 0.36	0.84 ± 0.29	0.83 ± 0.26	mg/dL	0.0799	0.0697
	ALP	201.33 ± 85.17	140.83 ± 48.51	166.83 ± 54.78	U/L	0.2366	0.596
	ALT	221 ± 280.86	114.33 ± 60.32	122.83 ± 101.92	U/L	0.4565	0.5106
	Total protein	6.65 ± 0.42	6.97 ± 0.57	6.6 ± 0.63	g/dL	0.4098	0.9759
	Albumin	3.3 ± 0.33	3.32 ± 0.44	3.1 ± 0.26	g/dL	0.9863	0.1888
	Globulin	3.35 ± 0.33	3.62 ± 0.25	3.52 ± 0.42	g/dL	0.4284	0.7054
	Total bilirubin	0.27 ± 0.13	0.48 ± 0.38	0.3 ± 0.17	mg/dL	0.1585	0.9864
	GGT	7.75 ± 6.60	10.25 ± 9.25	3.6 ± 4.39	U/L	0.8835	0.8429
	Total cholesterol	182.75 ± 39.96	182.25 ± 11.24	177.25 ± 15.41	mg/dL	0.9828	0.9286
	Phosphorus	3.47 ± 1.27	3.15 ± 0.95	4 ± 0.83	mg/dL	>0.9999	0.692
	Amylase	960.75 ± 976.14	507.5 ± 195.01	972.5 ± 877.26	U/L	0.518	0.9812
	Lipase	853.33 ± 120.37	711 ± 169.97	819.25 ± 226	U/L	0.8701	0.3355
	AST	47.4 ± 20.21	39.5 ± 8.27	40.4 ± 8.65	IU/I	0.4534	0.9132
	Triglycerides	64 ± 15.18	76.75 ± 24.16	89.4 ± 22.15	mg/dL	0.2938	0.2425
Electrolytes	Na^+^	149.83 ± 2.56	150 ± 1.41	151 ± 1.1	mmol/L	0.9844	0.4894
	K^+^	4.28 ± 0.35	4.2 ± 0.43	4.27 ± 0.31	mmol/L	0.621	0.981
	Ca^2+^	1.31 ± 0.06	1.36 ± 0.05	1.32 ± 0.03	mmol/L	0.1433	0.9586
	Cl^−^	115.67 ± 5.79	112.83 ± 3.87	115.33 ± 2.88	mmol/L	0.4141	0.9867

## 4 Discussion

Both the MSCs therapy group and the control group corresponded to MMVD stage B1. This study aimed to evaluate the safety and efficacy of gonadal tissue-derived mesenchymal stem cell therapy in canine patients with early stage canine MMVD. The MSC treatment group received five or more consecutive intravenous injections and cardiac monitoring was conducted at yearly intervals. The effects of treatment were assessed by comparison with a control group based on the progression to MMVD stage B2 and echocardiographic indicators. A statistically significant disparity was noted in the left atrial diameter (0.48 ± 2.86 mm in the MSC group and 2.97 ± 2.38 mm in the control group) and E-velocity (−0.08 ± 0.16 m/s in the MSC group and 0.26 ± 0.30 m/s in the control group) between the two groups (*p* < 0.05), indicating a favorable impact of MSC derived from the gonadal tissue on left atrial pressure. Additionally, the median progression duration to MMVD stage B2 was 730 days for the control group and 1,467 days for the MSC group. The treatment group demonstrated delayed progression (*p =* 0.038), enabling them to prolong their disease duration without requiring cardiac medication. This study revealed positive therapeutic effects in the MSC treatment group in MMVD B1 patients, with no significant adverse events were observed in the short- or long-term monitoring of blood indicators and reactions.

MMVD involves the occurrence of myxomatous degeneration in the mitral valves, which leads to valve fibrosis, morphological changes, and the onset of blood regurgitation, ultimately resulting in heart failure (25). Diego et al. analyzed the proinflammatory and immunological profiles to elucidate the pathophysiological mechanisms of canine MMVD. They found that Treg cells played a role in maintaining peripheral tolerance, and TNF-*α*, IL-1*β*, and IL-6 levels significantly increased with the severity of the disease in MMVD. A positive correlation between IL-6 and left ventricular diastolic volume suggests that inflammatory activation may be involved in cardiac remodeling related to progressive volumetric overload in MMVD (26). Moreover, a study stated that the transforming growth factor-*β* (TGF-β) signaling and reactive oxygen species significantly contribute to profibrotic gene expression in myxomatous mitral valves. They suggested that therapies targeting the reduction of TGF-β activation and oxidative stress in early MMVD might help decelerate its progression (4).

Mesenchymal stem cells are known for their ability to regenerate damaged tissues and demonstrate significant anti-inflammatory and antifibrotic effects. Notably, mesenchymal stem cell therapy suppresses profibrotic genes in experimental models of liver fibrosis by inhibiting the release of TGF- β (27). By leveraging redox systems, mesenchymal stem cell therapy has shown potential as an antifibrotic intervention to resist reactive oxygen species-induced oxidative stress (28). Patients with acute myocardial infarction demonstrate myocardial protection through mesenchymal stem cell therapy, which notably reduces inflammation, encourages myocardial cell differentiation and angiogenesis in infarct areas, enhances resistance to apoptosis, and hampers fibrosis (29). A limitation of this study is the inability to conduct ELISA tests for anti-inflammatory and anti-fibrotic markers on serum samples from actual patients. Therefore, future research should aim to address this issue.

In this study, we observed a deterioration in indicators in the control group from echocardiography conducted annually, whereas the mesenchymal stem cell therapy group showed overall maintenance of the indicators. The statistical differences observed in LA diameter and E-peak compared to the control group suggest a positive effect on left atrial pressure. It has been hypothesized that mesenchymal stem cell therapy in MMVD alleviates inflammation and fibrosis of the valve, which lowers the degree of blood reflux and, subsequently, left atrial pressure. Ultimately, it is suggested that these effects lead to a delay in the progression to stage B2 owing to the application of mesenchymal stem cell therapy in the early stages of MMVD.

The administration of mesenchymal stem cells to patients with early stage MMVD offers two key benefits. First, no specific treatment, including medication, is prescribed for MMVD stage B1; regular monitoring is primarily recommended (6). Conversely, based on research involving pimobendan in patients with MMVD stage B2, a decrease in left atrial pressure can be expected with recommended drug therapy (6). Therefore, mesenchymal stem cell therapy may be considered as a preemptive treatment for patients who have not yet been treated.

MSC therapy, when administered during the early stages of MMVD, effectively slows disease progression. This intervention holds promise, as it potentially avoids the side effects associated with multidrug therapy, including diuretics used in heart failure. Diuretics are essential in patients with heart failure. However, they can also lead to renal disease. Late-stage heart failure, they may even trigger a crisis called cardiorenal syndrome (CRS) (30, 31). Veterinarians face a treatment dilemma when managing heart and kidney diseases, which often result in a higher likelihood of mortality (32). By delaying the progression of heart disease and thereby reducing dependence on heart medications, MSC therapy presents a promising avenue for enhancing the quality of life and extending the survival time of patients with MMVD.

High-cost mesenchymal stem cell therapies may be unattainable for MMVD stage B1 who do not require extensive treatment. However, intravenous MSC administration is a suitable option for treating multiple chronic conditions due to the ability of MSCs to disseminate throughout the body (30). For older adult animals with several diseases, mesenchymal stem cell therapy offers not only heart-related benefits but also potential improvements across different diseases. The participants in this study had multiple comorbidities, and mesenchymal stem cell therapy was considered for its potential efficacy. Repeated MSC treatments are safe for older dogs with multiple health issues, including heart disease.

Determining an appropriate administration route is crucial in mesenchymal stem cell therapy depending on the characteristics of the disease (33). Various routes, such as intracoronary, intramyocardial injection, IV, and patch forms, have been studied for mesenchymal stem cell applications in heart diseases (15, 34–37). However, overly aggressive routes may not be advisable because of concerns about cardiac deterioration due to anesthesia or stress in patients with heart disease (38, 39). We demonstrated that a less invasive approach, such as IV administration, may offer therapeutic benefits to patients. Although IV administration may have lower engraftment rates than more aggressive methods, it is presumed to have positive effects on the heart due to the paracrine effects of the mesenchymal stem cell secretome (18). In another study, mesenchymal stem cells that migrated to the lungs of patients with myocardial infarction were redistributed to damaged heart tissue through homing effects (34).

Previous studies of IV MSC therapy in patients with MMVD found it challenging to observe long-term improvements in cardiac function (17, 18). One study showed a decline in results at 60 days compared to 30 days of mesenchymal stem cell therapy, raising concerns about the low survival rates of mesenchymal stem cells (17). In our study, IV mesenchymal stem cell therapy was conducted at 1-month intervals for over five sessions, with some patients receiving up to 12 sessions, resulting in improvements in echocardiographic indicators and a delay in progression to stage B2. This suggests that multiple treatment sessions over time could be more beneficial than a single treatment for patients with MMVD.

A major advantage of gonadal tissue-derived MSCs is that they can utilize the tissue discarded during neutering surgery in young, healthy animals. By collecting tissues from young animals and isolating autologous mesenchymal stem cells preserved through cryopreservation, a cytobanking operation can be established. This allows for the future use of these gonadal tissue-derived mesenchymal stem cells when animals reach an older age, when chronic diseases may develop. Jeung et al. reported on the safety of gonadal tissue-derived MSC therapy in geriatric dogs with chronic disease. Additionally, their study indicated that these results support the potential of gonadal tissue-derived MSCs as an effective therapeutic option (40). Through the MSCs’ characterization process, we confirmed that gonadal tissue is a source of MSCs. We demonstrated their safety and efficacy in early-stage MMVD patients.

Although this study included only six dogs in the MSC therapy group, future large-scale studies comparing the effects of mesenchymal stem cell therapy at different MMVD stages are needed to assess its efficacy. Moreover, because of the retrospective nature of the study, which was based on the treatment records of patients, mesenchymal stem cell migration or postmortem biopsies could not be tracked.

In our study, the control group included MMVD B1 patients who received standard management without pharmacological intervention until progression to B2. Due to the retrospective nature of the study, we were unable to implement a placebo treatment to control for potential biases. However, imaging parameters showed no significant differences between the two groups at baseline, indicating that they were comparable before treatment. The differences observed at the endpoint suggest that the potential influence of psychological effects was minimized.

Previous human studies have shown that MSC therapy provides economic benefits in the treatment of chronic and severe diseases. Notably, these studies highlight that MSC therapy can be cost-effective by delaying disease progression, improving quality of life and survival, reducing in-hospital mortality, and increasing discharge rates (41–43). While economic evaluations of MSC therapy have been conducted in human studies, no research has yet explored its economic benefits in canine MMVD, underscoring the need for further investigation in this area.

In conclusion, therapy with MSCs derived from gonadal tissue significantly delayed MMVD progression by maintaining the early-stage for a longer period. Therefore, MSC therapy is considered a safe and effective treatment for patients with MMVD stage B1 who are managed without treatment. Furthermore, the broad applicability of mesenchymal stem cells under complex conditions suggests their wide potential in veterinary medicine.

## Data Availability

The raw data supporting the conclusions of this article will be made available by the authors, without undue reservation.

## References

[1] Atkins C Bonagura J Ettinger S Fox P Gordon S Haggstrom J. Guidelines for the diagnosis and treatment of canine chronic valvular heart disease. J Vet Intern Med. (2009) 23:1142–50. doi: 10.1111/j.1939-1676.2009.0392.x, PMID: 19780929

[2] Kim H-T Han S-M Song W-J Kim B Choi M Yoon J. Retrospective study of degenerative mitral valve disease in small-breed dogs: survival and prognostic variables. J Vet Sci. (2017) 18:369–76. doi: 10.4142/jvs.2017.18.3.369, PMID: 28057898 PMC5639090

[3] Keene BW Atkins CE Bonagura JD Fox PR Häggström J Fuentes VL. Acvim consensus guidelines for the diagnosis and treatment of Myxomatous mitral valve disease in dogs. J Vet Intern Med. (2019) 33:1127–40. doi: 10.1111/jvim.15488, PMID: 30974015 PMC6524084

[4] Hagler MA Hadley TM Zhang H Mehra K Roos CM Schaff HV. Tgf-Β signalling and reactive oxygen species drive fibrosis and matrix remodelling in myxomatous mitral valves. Cardiovasc Res. (2013) 99:175–84. doi: 10.1093/cvr/cvt083, PMID: 23554457 PMC3687751

[5] Borgarelli M Haggstrom J. Canine degenerative myxomatous mitral valve disease: natural history, clinical presentation and therapy. Vet Clin North Am Small Anim Pract. (2010) 40:651–63. doi: 10.1016/j.cvsm.2010.03.008, PMID: 20610017

[6] Boswood A Häggström J Gordon SG Wess G Stepien RL Oyama MA. Effect of pimobendan in dogs with preclinical myxomatous mitral valve disease and cardiomegaly: the Epic study-a randomized clinical trial. J Vet Intern Med. (2016) 30:1765–79. doi: 10.1111/jvim.14586, PMID: 27678080 PMC5115200

[7] Swedberg K Cleland J Dargie H Drexler H Follath F Komajda M. Guidelines for the diagnosis and treatment of chronic heart failure: executive summary (update 2005): the task force for the diagnosis and treatment of chronic heart failure of the European Society of Cardiology. Eur Heart J. (2005) 26:1115–40. doi: 10.1093/eurheartj/ehi204, PMID: 15901669

[8] Kolf CM Cho E Tuan RS. Mesenchymal stromal cells: biology of adult mesenchymal stem cells: regulation of niche, self-renewal and differentiation. Arthritis Res Ther. (2007) 9:204. doi: 10.1186/ar2116, PMID: 17316462 PMC1860068

[9] Zhang R Liu Y Yan K Chen L Chen X-R Li P. Anti-inflammatory and immunomodulatory mechanisms of mesenchymal stem cell transplantation in experimental traumatic brain injury. J Neuroinflammation. (2013) 10:871. doi: 10.1186/1742-2094-10-106, PMID: 23971414 PMC3765323

[10] Wang M Yuan Q Xie L. Mesenchymal stem cell-based immunomodulation: properties and clinical application. Stem Cells Int. (2018) 2018:3057624–12. doi: 10.1155/2018/3057624, PMID: 30013600 PMC6022321

[11] Jin Q-H Kim H-K Na J-Y Jin C Seon J-K. Anti-inflammatory effects of mesenchymal stem cell-conditioned media inhibited macrophages activation in vitro. Sci Rep. (2022) 12:4754. doi: 10.1038/s41598-022-08398-4, PMID: 35306509 PMC8934344

[12] Berebichez-Fridman R Montero-Olvera PR. Sources and clinical applications of mesenchymal stem cells: state-of-the-art review. Sultan Qaboos Univ Med J. (2018) 18:e264–77. doi: 10.18295/squmj.2018.18.03.002, PMID: 30607265 PMC6307657

[13] Voga M Adamic N Vengust M Majdic G. Stem cells in veterinary medicine—current state and treatment options. Front Vet Sci. (2020) 7:278. doi: 10.3389/fvets.2020.00278, PMID: 32656249 PMC7326035

[14] Quimby JM Borjesson DL. Mesenchymal stem cell therapy in cats: current knowledge and future potential. J Feline Med Surg. (2018) 20:208–16. doi: 10.1177/1098612x18758590, PMID: 29478398 PMC10816289

[15] Hensley MT Tang J Woodruff K Defrancesco T Tou S Williams CM. Intracoronary allogeneic cardiosphere-derived stem cells are safe for use in dogs with dilated cardiomyopathy. J Cell Mol Med. (2017) 21:1503–12. doi: 10.1111/jcmm.13077, PMID: 28296006 PMC5543505

[16] Sousa MG Paulino-Junior D Pascon JP Pereira-Neto GB Carareto R Champion T. Cardiac function in dogs with chronic Chagas cardiomyopathy undergoing autologous stem cell transplantation into the coronary arteries. Can Vet J. (2011) 52:869–74. PMID: 22294793 PMC3135031

[17] Petchdee S Sompeewong S. Intravenous administration of puppy deciduous teeth stem cells in degenerative valve disease. Vet World. (2016) 9:1429–34. doi: 10.14202/vetworld.2016.1429-1434, PMID: 28096616 PMC5234058

[18] Yang VK Meola DM Davis A Barton B Hoffman AM. Intravenous administration of allogeneic Wharton jelly-derived mesenchymal stem cells for treatment of dogs with congestive heart failure secondary to Myxomatous mitral valve disease. Am J Vet Res. (2021) 82:487–93. doi: 10.2460/ajvr.82.6.487, PMID: 34032485 PMC8224903

[19] Kabat M Bobkov I Kumar S Grumet M. Trends in mesenchymal stem cell clinical trials 2004-2018: is efficacy optimal in a narrow dose range? Stem Cells Transl Med. (2020) 9:17–27. doi: 10.1002/sctm.19-0202, PMID: 31804767 PMC6954709

[20] Penha EM Meira CS Guimarães ET Mendonça MV Gravely FA Pinheiro CM. Use of autologous mesenchymal stem cells derived from bone marrow for the treatment of naturally injured spinal cord in dogs. Stem Cells Int. (2014) 2014:437521:1–8. doi: 10.1155/2014/437521, PMID: 24723956 PMC3956412

[21] Vikartovska Z Kuricova M Farbakova J Liptak T Mudronova D Humenik F. Stem cell conditioned medium treatment for canine spinal cord injury: pilot feasibility study. Int J Mol Sci. (2020) 21:5129. doi: 10.3390/ijms21145129 32698543 PMC7404210

[22] Rhew SY Park SM Li Q An JH Chae HK Lee JH. Efficacy and safety of allogenic canine adipose tissue-derived mesenchymal stem cell therapy for insulin-dependent diabetes mellitus in four dogs: a pilot study. J Vet Med Sci. (2021) 83:592–600. doi: 10.1292/jvms.20-0195, PMID: 33551441 PMC8111340

[23] Connick P Kolappan M Crawley C Webber DJ Patani R Michell AW. Autologous mesenchymal stem cells for the treatment of secondary progressive multiple sclerosis: an open-label phase 2a proof-of-concept study. Lancet Neurol. (2012) 11:150–6. doi: 10.1016/s1474-4422(11)70305-2, PMID: 22236384 PMC3279697

[24] Lavan RP. Development and validation of a survey for quality of life assessment by owners of healthy dogs. Vet J. (2013) 197:578–82. doi: 10.1016/j.tvjl.2013.03.021, PMID: 23639368

[25] Oyama MA Elliott C Loughran KA Kossar AP Castillero E Levy RJ. Comparative pathology of human and canine Myxomatous mitral valve degeneration: 5ht and Tgf-Β mechanisms. Cardiovasc Pathol. (2020) 46:107196. doi: 10.1016/j.carpath.2019.107196, PMID: 32006823 PMC7078050

[26] Piantedosi D Musco N Palatucci AT Carriero F Rubino V Pizzo F. Pro-inflammatory and immunological profile of dogs with Myxomatous mitral valve disease. Vet Sci. (2022) 9:326. doi: 10.3390/vetsci9070326, PMID: 35878343 PMC9315642

[27] Hermansyah D Putra A Muhar AM Retnaningsih WK Dirja BT. Mesenchymal stem cells suppress Tgf-Β release to decrease Α-Sma expression in ameliorating Ccl4-induced liver fibrosis. Med Arch. (2021) 75:16–22. doi: 10.5455/medarh.2021.75.16-22, PMID: 34012193 PMC8116080

[28] Kumar S Verma R Tyagi N Gangenahalli G Verma YK. Therapeutics effect of mesenchymal stromal cells in reactive oxygen species-induced damages. Hum Cell. (2022) 35:37–50. doi: 10.1007/s13577-021-00646-5, PMID: 34800267 PMC8605474

[29] Guo Y Yu Y Hu S Chen Y Shen Z. The therapeutic potential of mesenchymal stem cells for cardiovascular diseases. Cell Death Dis. (2020) 11:349. doi: 10.1038/s41419-020-2542-9, PMID: 32393744 PMC7214402

[30] Chen J Luo L Tian R Yu C. A review and update for registered clinical studies of stem cells for non-tumorous and non-hematological diseases. Regenerative Therapy. (2021) 18:355–62. doi: 10.1016/j.reth.2021.09.001, PMID: 34584912 PMC8446785

[31] Casu G Merella P. Diuretic therapy in heart failure - current approaches. Eur Cardiol. (2015) 10:42–7. doi: 10.15420/ecr.2015.10.01.42, PMID: 30310422 PMC6159465

[32] Taveira Gomes T Santos Araujo C Valente F Bernardo F Seabra Carvalho D Bodegard J. Cardiorenal syndrome and death risk in patients with heart failure or chronic kidney disease: an unmet cardiorenal need? Eur Heart J. (2021) 42:819. doi: 10.1093/eurheartj/ehab724.0819

[33] Caplan H Olson SD Kumar A George M Prabhakara KS Wenzel P. Mesenchymal stromal cell therapeutic delivery: translational challenges to clinical application. Front Immunol. (2019) 10:10. doi: 10.3389/fimmu.2019.01645, PMID: 31417542 PMC6685059

[34] Campbell NG Suzuki K. Cell delivery routes for stem cell therapy to the heart: current and future approaches. J Cardiovasc Transl Res. (2012) 5:713–26. doi: 10.1007/s12265-012-9378-3, PMID: 22648235

[35] Yagyu T Yasuda S Nagaya N Doi K Nakatani T Satomi K. Long-term results of intracardiac mesenchymal stem cell transplantation in patients with cardiomyopathy. Circ J. (2019) 83:1590–9. doi: 10.1253/circj.CJ-18-1179, PMID: 31105128

[36] Wang QL Wang HJ Li ZH Wang YL Wu XP Tan YZ. Mesenchymal stem cell-loaded cardiac patch promotes epicardial activation and repair of the infarcted myocardium. J Cell Mol Med. (2017) 21:1751–66. doi: 10.1111/jcmm.13097, PMID: 28244640 PMC5571540

[37] Lee K Xue Y Lee J Kim HJ Liu Y Tebon P. A patch of detachable hybrid microneedle depot for localized delivery of mesenchymal stem cells in regeneration therapy. Adv Funct Mater. (2020) 30:2000086. doi: 10.1002/adfm.202000086, PMID: 33071712 PMC7567343

[38] Froehlich JB Eagle KA. Anaesthesia and the cardiac patient: the patient versus the procedure. Heart. (2002) 87:91–6. doi: 10.1136/heart.87.1.91, PMID: 11751677 PMC1766966

[39] Hughes JML. Anaesthesia for the geriatric dog and cat. Ir Vet J. (2008) 61:380–7. doi: 10.1186/2046-0481-61-6-380, PMID: 21851715 PMC3113863

[40] Jeung S-Y An J-H Kim S-S Youn H-Y. Safety of gonadal tissue-derived mesenchymal stem cell therapy in geriatric dogs with chronic disease. Animals. (2024) 14:2134. doi: 10.3390/ani14142134, PMID: 39061596 PMC11273526

[41] Suh K Cole BJ Gomoll A Lee SM Choi H Ha CW. Cost effectiveness of allogeneic umbilical cord blood-derived mesenchymal stem cells in patients with knee osteoarthritis. Appl Health Econ Health Policy. (2023) 21:141–52. doi: 10.1007/s40258-022-00762-9, PMID: 36136263 PMC9834379

[42] Barry LE Crealey GE Cockwell P Elliman SJ Griffin MD Maxwell AP. Mesenchymal stromal cell therapy compared to Sglt2-inhibitors and usual Care in Treating Diabetic Kidney Disease: a cost-effectiveness analysis. PLoS One. (2022) 17:e0274136. doi: 10.1371/journal.pone.0274136, PMID: 36331936 PMC9635741

[43] Thavorn K van Katwyk S Krahn M Mei SHJ Stewart DJ Fergusson D. Value of mesenchymal stem cell therapy for patients with septic shock: an early health economic evaluation. Int J Technol Assess Health Care. (2020) 36:525–32. doi: 10.1017/S0266462320000781 33059782

